# ﻿Two new species of the genus *Norellisoma* Wahlgren, 1917 (Diptera, Scathophagidae) from Yintiaoling Nature Reserve, Chongqing, China

**DOI:** 10.3897/zookeys.1168.104151

**Published:** 2023-06-27

**Authors:** Xiao-Dong Cai, Xin Li, Wen-Qiang Cao, Ding Yang

**Affiliations:** 1 Department of Entomology, College of Plant Protection, China Agricultural University, Beijing 100193, China China Agricultural University Beijing China

**Keywords:** Dung fly, identification key, morphology, new taxon, taxonomy

## Abstract

The known species of the genus *Norellisoma* from China are reviewed and two new species from Yintiaoling Nature Reserve in Chongqing City, where no other *Norellisoma* species are recorded, are described: *Norellisomawuxiense***sp. nov.**, *Norellisomayintiaoense***sp. nov.** A key to the species of *Norellisoma* from China is provided.

## ﻿Introduction

The genus *Norellisoma* Wahlgren, 1917 is a moderately large genus of the Scathophagidae distributed in the northern hemisphere, reaching its highest diversity in the Palaearctic Region (Ozerov and Krivosheina 2020; Iwasa and Sasaki 2022). At the present time, the genus comprises 29 species, four of which have been recorded in China (Sun 1992; Ozerov 2009; Bernasconi and Šifner 2021; Iwasa and Sasaki 2022).

Yintiaoling Nature Reserve is located in northeast Chongqing City, whose average elevation is 1900 m asl. In Chongqing, Yintiaoling National Nature Reserve is the only primary forest with rich species diversity but the fauna of *Norellisoma* is poorly known. In our paper, we describe and illustrate two new species from Yintiaoling National Nature Reserve. A key to the six known species from China is presented (Xue and Zhao 1996).

## ﻿Material and methods

Specimens were studied and illustrated with Olympus SZ61 stereoscope. The specimens were photographed with a Canon EOS 5Ds camera and stacked with Helicon Focus Software and imported into Adobe Photoshop CC2020 for labelling and plate composition. Genitalia were prepared by macerating the apical portion of the abdomen in warm 10% NaOH for 17–20 minutes, and then washing in distilled water. After examination, genitalia were transferred to fresh glycerine and stored in microvials pinned below the specimens. All the examined specimens, including holotypes and paratypes, are deposited in the China Agricultural University in Beijing (CAU). Morphological terminology follows Cumming and Wood (2017). The following abbreviations are used: ***fr s*** = frontal seta(e), ***orb s*** = orbital seta(e), ***oc s*** = ocellar seta(e), ***i vt s*** = inner vertical seta(e), ***o vt s*** = outer vertical seta(e), ***poc s*** = postocellar seta(e), ***dc s*** = dorsocentral seta(e), ***ial s*** = intra-alar seta(e), ***spal s*** = supra-alar seta(e), ***pprn s*** = postpronotal seta(e), ***npl s*** = notopleural seta(e), ***pal s*** = postalar seta(e), ***a*** = anterior seta(e), ***ad*** = anterodorsal seta(e), ***av*** = anteroventral seta(e), ***d*** = dorsal seta(e), ***p*** = posterior seta(e), ***pd*** = posterodorsal seta(e), ***pv*** = posteroventral seta(e), ***v*** = ventral seta(e), **epiph** = epiphallus, **pgt** = postgonite, **distph** = distiphallus, **pregt** = pregonite, **epand** = epandrium, **sur** = surstylus, **cerc** = cercus, **st4** = sternite 4, **st5** = sternite 5.

## ﻿Taxonomy

### ﻿Genus Norellisoma

Taxon classificationAnimaliaDipteraScathophagidae

Wahlgren, 1917

F28B8E7E-2C2F-52A5-8696-759C36C0B4DE

Norellisoma Hendel, 1910: 308. Nomen nudum.
Norellisoma Wahlgren, 1917: 148. Type species: Cordyluraspinimana Fallén, 1819 (subsequent designation by Vockeroth 1965).

### ﻿Key to the species of *Norellisoma* from China

**Table:** 

1	Scutum mostly grayish yellow	**2**
–	Scutum usually yellow with two blackish stripes along dorsocentral lines	**4**
2	Legs entirely yellow, but mid and hind femora usually blackish dorsally; abdomen with yellowish brown pruinescence	***N.armipes* (Meigen, 1826)**
–	Mid and hind femora with a dark streak dorsally; abdomen with pale gray pruinescence	**3**
3	Male sternite 5 with adjacent lateral lobes	***N.striolatum* (Meigen, 1826)**
–	Male sternite 5 with triangular lateral lobes usually strong divergent	***N.triangulum* (Sun, 1992)**
4	Male sternite 4 as wide as long	***N.spinimanum* (Fallén, 1819)**
–	Male sternite 4 wider than long	**5**
5	Pregonite with 3 brown setae; postgonite dorsally curved	***N.wuxiense* sp. nov.**
–	Pregonite with 3 yellow setae; postgonite apically curved	***N.yintiaoense* sp. nov.**

### ﻿ Norellisoma wuxiense sp. nov.

Taxon classificationAnimaliaDipteraScathophagidae

A0595704-60AE-5775-BB61-A4268164654D

https://zoobank.org/E8F057B4-E7ED-4ADC-8DBF-1D07B6807CBA

Figs 1 , 2 , 5–9

#### Type material.

***Holotype***: ♂, **China**: Chongqing: Wuxi, Yintiaoling Nature Reserve, 31°28'15"N, 109°56'33"E; 2939 m a.s.l.; 14. viii. 2022, leg. Xulong Chen (CAU); CAU(H)202398101. ***Paratypes***: 4♂♂ 4♀♀, same data as holotype (CAU); CAU(P)202398102 – CAU(P)202398109.

#### Diagnosis.

Thorax yellow, scutum along dorsocentral setae with blackish stripe. Male sternite 4 rectangular, wider than long. Male sternite 5 with diverging lateral lobes and two short median projections. Pregonite with 3 brown setae; postgonite dorsally curved.

#### Description.

**Figure 1.**
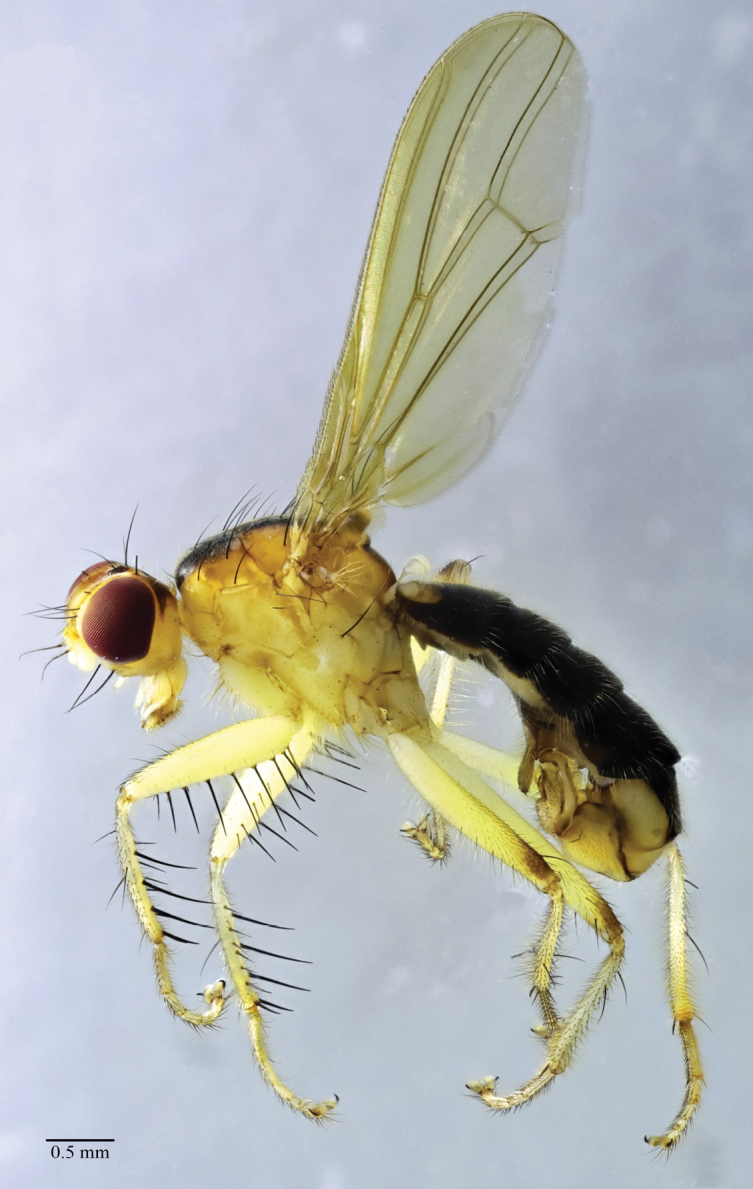
*Norellisomawuxiense* sp. nov. (holotype, male). Habitus, lateral view. Scale bar: 0.5 mm.

**Figure 2.**
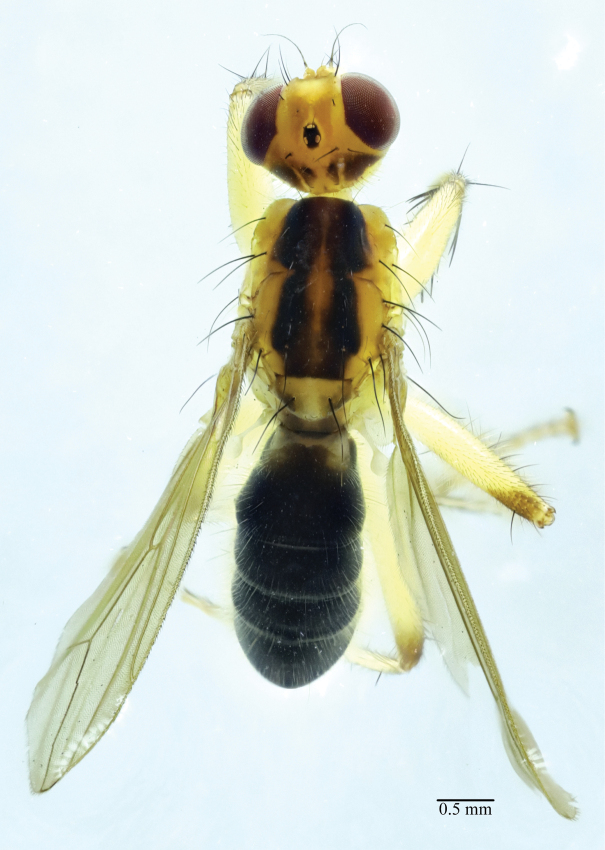
*Norellisomawuxiense* sp. nov. (holotype, male). Habitus, dorsal view. Scale bar: 0.5 mm.

**Male** (Figs 1, 2). Body length 5.77–6.33 mm, wing length 5.17–5.33 mm.

***Head*** yellow. Eyes reddish brown, occiput yellow to black. Setulae on head yellow, setae brown to black; 3–4 *fr s*, 2 *orb s*, 1 *oc s*, 1 *i vt s*, 1 *o vt s*, 1 *poc s*. 1 pair of strong vibrissae. Antenna yellow except arista yellow to blackish brown, first flagellomere 2 times as long as wide; arista with short blackish brown pubescence. Proboscis yellow; palpus yellow with yellow setulae and setae.

***Thorax*** yellow, scutum along dorsocentral setae with blackish stripe. Hairs on thorax yellow, setae black. 2+3 *dc s*, 1+2 *spal s*, 1 *pprn s*, 1 *npl s*, 2 *pal s*. Proepisternum with 1 yellowish brown seta. Anepisternum with 1 long seta near posterior margin. Katepisternum with 1 seta in posterodorsal corner. Scutellum with one pair of strong setae.

***Legs*** mostly yellow, but mid and hind femora usually blackish dorso-distally. Setulae on legs yellow, setae black or yellowish brown. Fore femur with row of 8 short *av* and 10 long *pv.* Fore tibia with row of 7 *av* and 4 *pv*, also with 1 *ad*, 1 *d*, 1 *pd* at apex. Mid femur with 1 *pd* and row of 5 short *a.* Mid tibia with 1 *ad*, 1 *pd* and 1 *av*, also with 1 *ad*, 1 *d*, 1 *pd*, 1 *v*, 1 *pv* at apex. Hind femur with 1 *v* and row of 5 short *a.* Hind tibia with 3 *ad*, 1 *d*, 2 *pd* and 1 *av* at apex.

***Wings*** hyaline; veins brown. R_1_ bare; CuA_2_+ A_1_ not extending to wing margin. Calypter yellow. Halteres yellowish white.

***Abdomen*** blackish brown. Setulae and setae yellow. Male sternite 4 rectangular, wider than long. Male sternite 5 with diverging lateral lobes and two short median projections.

***Male genitalia*** (Figs 5–9): Epandrium yellowish brown with hair-like setulae; surstylus curved inward and antero-apically broadened slightly; cercus well broadened, covered with black setulae; distiphallus dorsally sclerotized and apically slender; pregonite broad, dorsally curved with 3 brown setae; postgonite rod-shaped, dorsally curved.

**Female**: Body length 6.0–7.0 mm, wing length 5.0–6.0 mm.

#### Distribution.

China (Chongqing).

#### Etymology.

The specific epithet refers to the type locality Wuxi.

#### Remarks.

This new species is similar to *Norellisomaspinimanum* (Fallén, 1819), but may be separated from the latter by the following features: male sternite 4 rectangular, wider than long; male sternite 5 with diverging lateral lobes and two short median projections.

### ﻿ Norellisoma yintiaoense sp. nov.

Taxon classificationAnimaliaDipteraScathophagidae

5C1E58F6-1333-5990-95CD-E9B149D56E20

https://zoobank.org/AAAAD140-3AC2-407D-AA60-BB7EC6A35544

Figs 3 , 4 , 10–14

#### Type material.

***Holotype***: ♂, **China**: Chongqing: Wuxi, Yintiaoling Nature Reserve, 31°28'15"N, 109°56'33"E; 2385 m a.s.l.; 22. vii. 2022, leg. Xulong Chen (CAU); CAU(H)202398201. ***Paratypes***: 1♂ 2♀♀, same data as holotype (CAU); CAU(P)202398202 – CAU(P)202398204.

#### Diagnosis.

Thorax yellow, scutum along dorsocentral setae with blackish stripe. Male sternite 4 rectangular, wider than long. Male sternite 5 with long narrow nearly parallel lateral lobes and two short median projections. Pregonite with 3 yellow setae; postgonite apically curved.

#### Description.

**Figure 3.**
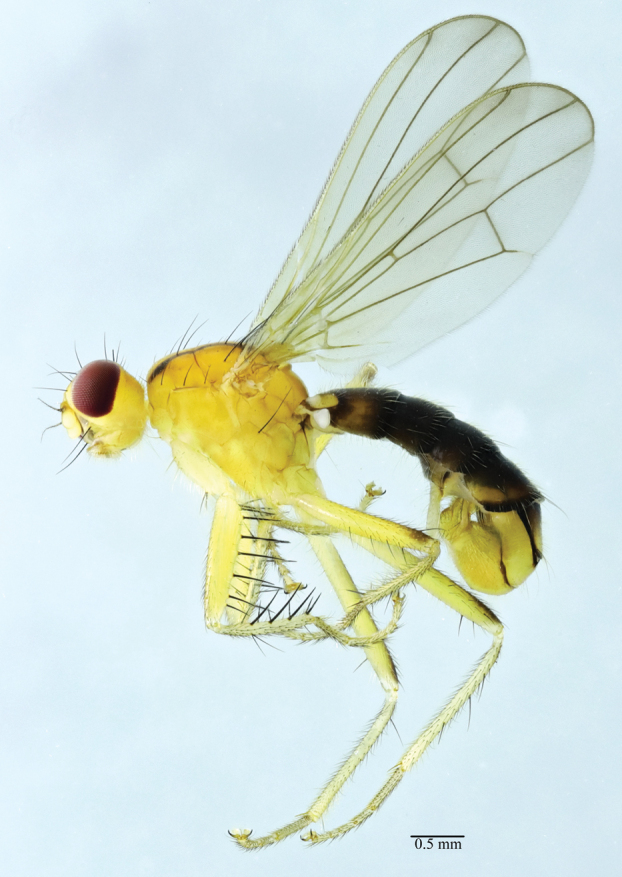
*Norellisomayintiaoense* sp. nov. (holotype, male). Habitus, lateral view. Scale bar: 0.5 mm.

**Figure 4.**
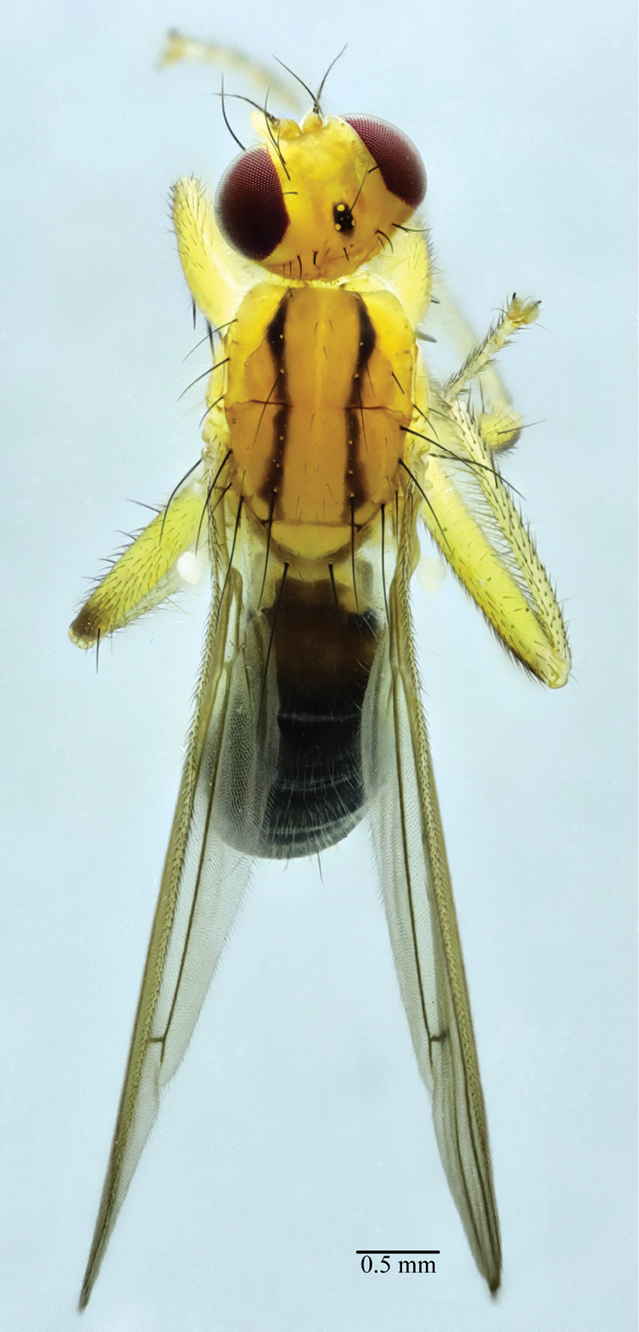
*Norellisomayintiaoense* sp. nov. (holotype, male). Habitus, dorsal view. Scale bar: 0.5 mm

**Male** (Figs 3, 4) Body length 5.33–6.00 mm, wing length 4.33–5.50 mm.

***Head*** yellow. Eyes reddish brown. Setulae on head yellow, setae black; 3 *fr s*, 2 *orb s*, 1 *oc s*, 1 *i vt s*, 1 *o vt s*, 1 *poc s*. 1 pair of strong vibrissae. Antenna yellow except arista yellow to blackish brown, first flagellomere 2 times as long as deep; arista with short blackish brown pubescence. Proboscis yellow; palpus yellow with yellow setulae and setae.

**Figures 5–9.**
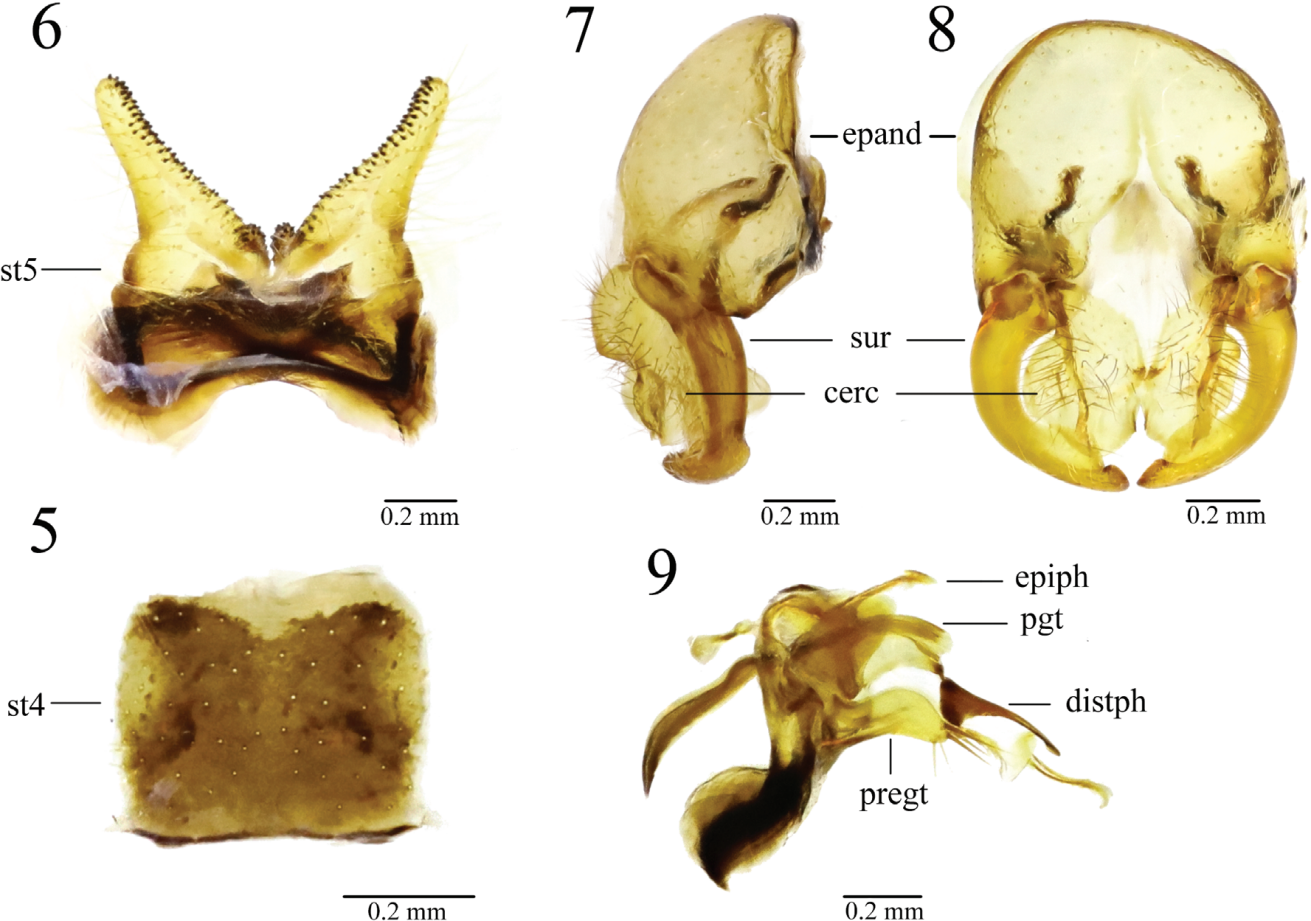
*Norellisomawuxiense* sp. nov. **5** male sternite 4, ventral view **6** male sternite 5, ventral view **7** male terminalia, lateral view **8** male terminalia, posterior view **9** aedeagus and associated parts, lateral view. Epiph = epiphallus, pgt = postgonite, distph = distiphallus, pregt = pregonite, epand = epandrium, sur = surstylus, cerc = cercus, st4 = sternite 4, st5 = sternite 5. Scale bars: 0.2 mm.

***Thorax*** yellow, scutum along dorsocentral setae with blackish stripe. Hairs on thorax yellow, setae black. 2+3 *dc s*, 1+2 *spal s*, 1 *pprn s*, 2 *npl s*, 2 *pal s*. Proepisternum with 1 yellowish brown seta. Anepisternum with 3 long setae near posterior margin. Katepisternum with 1 seta in posterodorsal corner. Scutellum with one pair of strong setae.

***Legs*** mostly yellow, but mid and hind femora usually blackish dorso-distally. Setulae on legs yellow, setae black or yellowish brown. Fore femur with row of 5 short *av* and 8 long *pv.* Fore tibia with 1 *ad*, 1 *pd*, 5 *av*, 4 *pv* and 1 preapical *d.* Mid femur with 1 *pd*, 1 *av*, 3–4 *a.* Mid tibia with 1 *ad*, 1 *pd* and 2 *av*, also with 1 *d*, 1 *v*, 1 *pv* at apex. Hind femur with 2 *v* and row of 5 short *a.* Hind tibia with 2 *ad*, 1 *d*, 2 *pd* and 1 *av* at apex.

***Wings*** hyaline; veins brown. R_1_ bare; CuA_2_+ A_1_ not extending to wing margin. Calypter yellow. Halteres yellowish white.

***Abdomen*** blackish brown. Setulae and setae yellowish brown. Male sternite 4 rectangular, wider than long. Male sternite 5 with long narrow nearly parallel lateral lobes and two short median projections.

**Figures 10–14.**
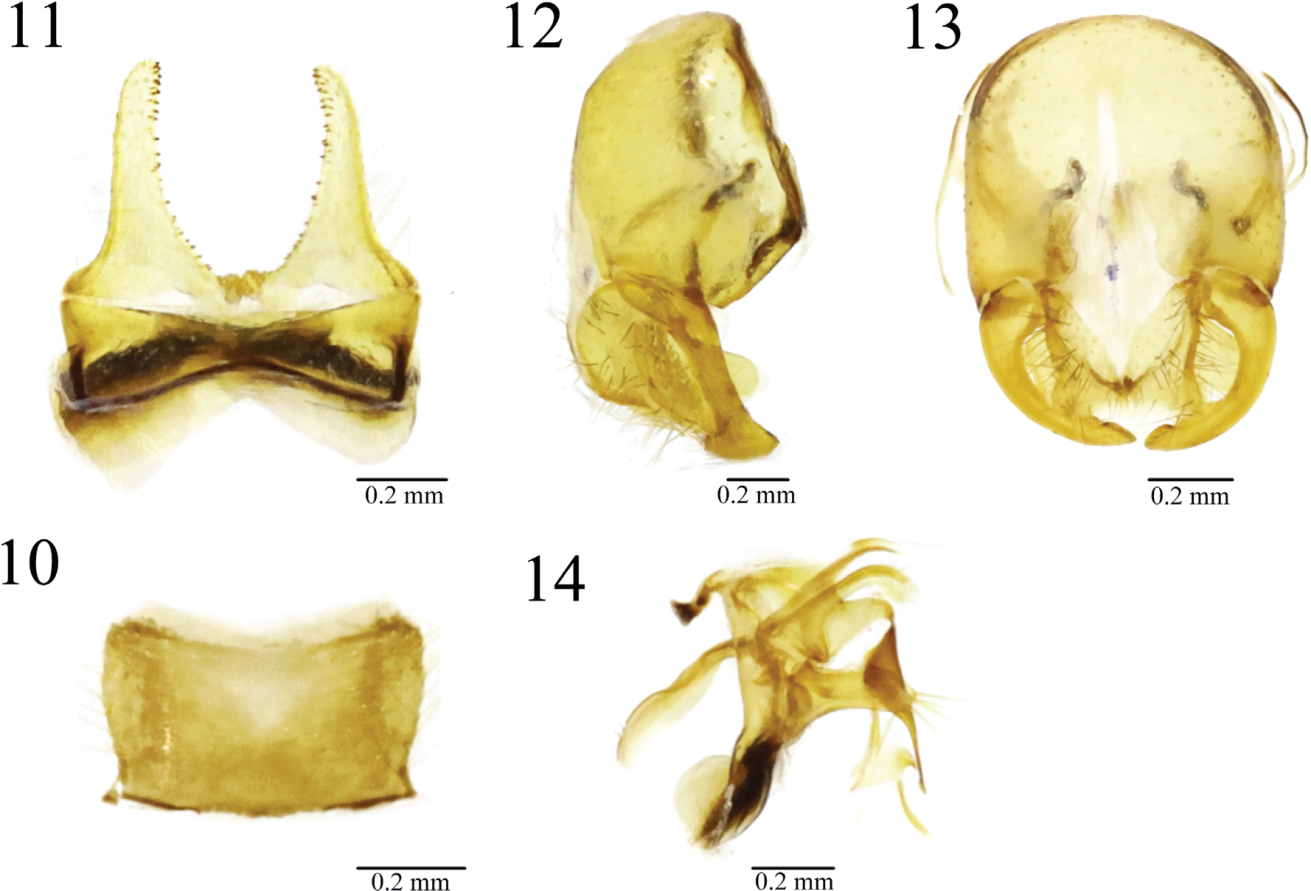
*Norellisomayintiaoense* sp. nov. **10** male sternite 4, ventral view **11** male sternite 5, ventral view **12** male terminalia, lateral view **13** male terminalia, posterior view **14** aedeagus and associated parts, lateral view. Scale bars: 0.2 mm.

***Male genitalia*** (Figs 10–14): Epandrium yellowish brown; surstylus curved inward and antero-apically broadened slightly; cercus well broadened, covered with black brown setulae; distiphallus dorsally sclerotized and apically slender; pregonite broad and apically with 3 yellow setae; postgonite rod-shaped, apically curved.

**Female**: Body length 6.50–6.67 mm, wing length 5.85–6.00 mm.

#### Distribution.

China (Chongqing).

#### Etymology.

The specific epithet refers to the type locality Yintiaoling Nature Reserve.

#### Remarks.

This new species is similar to *Norellisomawuxiense* sp. nov., but may be separated from the latter by the following features: pregonite not curved with 3 yellow apical setae and postgonite apically curved.

## ﻿Discussion

In this paper, we reported two new *Norellisoma* species from Yintiaoling Nature Reserve in Chongqing City. This is also the first time that *Norellisoma* species have been recorded in the Oriental region. Up to now, the genus *Norellisoma* comprised 31 known species globally. Among them, the following 28 species occur in the Palaeartic region: *N.alpestre* (Schiner, 1864); *N.armipes* (Meigen, 1826); *N.caucasicum* (Ozerov, 1993); *N.ezoensis* Iwasa & Sasaki, 2022; *N.femorale* (Loew, 1864); *N.flavicorne* (Meigen, 1826); *N.flavostriatum* Ozerov, 2008; *N.insulare* (Ozerov,1993); *N.ivanae* Šifner, 2003; *N.japonicum* Hironaga & Suwa, 2005; *N.jelineki* Šifner, 2006; *N.lesgiae* (Becker, 1894); *N.lituratum* (Meigen, 1826); *N.mireki* Šifner, 1977; *N.mirusae* Šifner, 1974; *N.montanopratense* (Ozerov, 1993); *N.nervosum* (Meigen, 1826); *N.nigrovenosum* Ozerov, 2008; *N.orientale* (Ozerov, 1993); *N.oreinum* Ozerov, 2010; *N.seguyi* Šifner, 1973; *N.striolatum* (Meigen, 1826); *N.sylviae* Šifner, 1999; *N.triangulum* (Sun, 1992); *N.tomkovichi* Ozerov, 2010; *N.vockerothi* Ozerov, 2013; *N.vonickai* Šifner, 2008; *N.yolduense* (Ozerov, 2008). The species *N.spinimanum* (Fallén, 1819) occurs both in the Nearctic (Canada, USA) and the Palaearctic regions, whereas the following six species are introduced to China, mainly distributed in Chongqing: *N.wuxiense* sp. nov., *N.yintiaoense* sp. nov., *N.spinimanum* (Jilin), *N.striolatum*, *N.armipes*, *N.triangulum* (latter two introduced to Sichuan and Xizang).

## Supplementary Material

XML Treatment for Norellisoma

XML Treatment for Norellisoma wuxiense

XML Treatment for Norellisoma yintiaoense

## ﻿Additional information

### Author ORCIDs

Xiao-Dong Cai https://orcid.org/0000-0002-2800-4244

Xin Li https://orcid.org/0000-0003-0773-6232

### Data availability

All of the data that support the findings of this study are available in the main text.
